# Electronic Nicotine Delivery Systems (ENDS), Marginalized Populations, and Tobacco Regulatory Policies

**DOI:** 10.29245/2689-999x/2023/2.1183

**Published:** 2023

**Authors:** Shervin Assari, Payam Sheikhattari

**Affiliations:** 1 Department of Urban Public Health, Charles R Drew University of Medicine and Science, Los Angeles, CA, USA; 2 Department of Family Medicine, Charles R Drew University of Medicine and Science, Los Angeles, CA, USA; 3 Department of Internal Medicine, Charles R Drew University of Medicine and Science, Los Angeles, CA, USA; 4 Center for Urban Health Disparities Research and Innovation, Morgan State University, Baltimore, MD, USA; 5 The Prevention Sciences Research Center, School of Community Health and Policy, Morgan State University, Baltimore, MD, USA

Electronic nicotine delivery systems (ENDS) including electronic cigarettes (e-cigarettes) deliver high doses of nicotine, a highly addictive drug^1^. A growing body of evidence suggests that nicotine use during adolescence and early adulthood increases the risk of nicotine dependence, poly-nicotine product use, the use of cannabis, and addiction to other drugs^2,3^. Nicotine and the other ingredients in e-cigarettes, many of which are toxic and carcinogenic, also have been linked to numerous adverse health effects, including cardiovascular and respiratory disease, cancer, and mental health and cognitive problems^4,5^.

The landscape of e-cigarettes and ENDS is undergoing rapid transformation, paralleled by swift changes in the market. To illustrate this transformation, ENDS initially emerged in the form of cig-a-likes, refillable systems, and tank setups, which relied on freebase nicotine that proved unappealing at higher nicotine concentrations. Subsequently, this product line evolved into pod mods and disposables employing nicotine salts. This shift in both design and functionality has played a role in reducing the perceived risk of e-cigarettes among adolescents^6-8^.

Due to the emergence of e-cigarettes and their strong appeal to adolescents and emerging adults, a generation that was on the cusp of being the first to broadly reject cigarette smoking and become tobacco free, instead has experienced nicotine dependence on e-cigarettes. This is in part due to inadequate regulatory oversight over the manufacture, marketing, and sale of e-cigarette products.

Due to the lower prevalence of e-cigarette use in racial and ethnic minority groups compared to White individuals.^9^, many scholars have avoided the term disparities for e-cigarette use across subpopulations^10-12^. We argue that there are still many reasons why we may think about e-cigarettes in terms of disparities framework.

First, economic inequalities correlate with disparities in e-cigarette use. In a 2023 study by Benny et al., an increased Gini coefficient was associated with daily e-cigarette use only in girls. The authors combined individual-level survey data from the Cannabis, Obesity, Mental Health, Physical Activity, Alcohol Use, Smoking, and Sedentary Behavior (COMPASS) study and area-level data from the Canadian Census (year 2016). Then, they applied multi-level logistic regression models to assess the relationship between income inequality and adolescent daily and current use of cannabis, cigarettes, and e-cigarettes. Their analytic sample was composed of a total of 74,501 students aged 12–19 who were 50.4% male and 69.1% White. While Gini was not significantly associated with daily e-cigarette use, the study documented a significant interaction between Gini and gender (Odds Ratio: OR=0.87, 95% CI= 0.80–0.94), indicating that increased income inequality was associated with higher risk of reporting daily e-cigarette use among females only^13^.

Second, there are racial and ethnic disparities in the following factors that shape e-cigarette use outcomes and exposures: (1) exposure to tobacco ads, regardless of product^14^, (2) uptake of tobacco control policies such as smoke-free zones^15^, (3) mistrust in tobacco control policies and related agencies such as FDA^16^, (4) trust in tobacco companies^17^, (5) care seeking^18^, (6) literacy and perceived risk^19^, (7) co-use^20^, (8) negative consequences such as dependence^21^ and related morbidity and mortality^22^, (9) susceptibility to nicotine dependence with lower level of exposure^23^, and (10) relapse after quitting^24^. A study by Truth initiative on seven hundred and fifty tobacco outlets in the Washington DC area showed a six times higher presence of tobacco advertisements on the exterior of gas stations than on other retail store types, and lower presence of advertisements at bars or restaurants that sold tobacco (OR=0.33), a spatial inequality that differentially exposed racial and ethnic groups to advertisement of various tobacco ads. Exterior tobacco ads are three times more likely in areas of the city that were predominantly African American^25-27^. Illicit sales of banned tobacco products based on age were more common for high schools in majority African American block groups (OR=1.29)^14^. According to one study, African American and Asian people had seven to nine times higher odds of trusting tobacco and e-cigarette companies regarding the information about the health effects of e-cigarettes than White people^17^. This may be because marginalized communities may have lower trust toward governmental institutes and organizations such as CDC and FDA^28,29^, due to previous experiences^30^. Analysis of the 2017 Youth Risk Behavior Survey (YRBS) showed that American Indian / Native American students had higher odds than White peers of being dual users (Relative Risk Ratio = RRR), 2.10, 95% CI, 1.01, 4.39), while African American, Hispanic, Asian and multi-racial and ethnic groups had lower odds than White peers of being dual users. Additionally, Asian students had lower odds than White students of being e-cigarette only users, whereas African American and Asian students had lower odds than their White peers of being cigarette only users. Also, American Indian/Alaskan Native students are most vulnerable to e-cigarette/cigarette use^20^.

Third, there already exists some evidence suggesting that marginalized groups based on race and ethnicity, sexual orientation, and sex may have a higher prevalence of e-cigarette use (Figure 1). There is an increased prevalence of e-cigarette use in American Indian/Alaskan Native individuals^31^. In the 2018-2019 Tobacco Use Supplement to the Current Population Survey, the largest nationally representative tobacco use survey of US adults, the prevalence of vaping was higher among men (2.8%; 95% CI, 2.7%-3.0%) and among non-Hispanic White (2.8%), American Indian/Alaskan Native (4.2%), and multiracial (4.5%) individuals^31^. In addition, there is a higher prevalence of e-cigarette use in high SES racial and ethnic minorities than high SES non-Hispanic White people^32^. In addition, African American people show a lower prevalence of tobacco use in early life than their white counterparts, but face a larger increase in the prevalence of tobacco use in adulthood. This pattern is probably due to African American adults facing blocked opportunities related to employment and living a prosperous life^33^. In addition, the decline in tobacco use among youths is showing a larger rate in White than African American youths, according to Monitoring the Future^34^. Finally, as Healthy People 2030 suggested^35^, African American people tend to use tobacco for longer periods before attempting to quit and make more quit attempts, and at the same time are less successful in quitting tobacco than non-Hispanic White people^36^. A study showed African American communities’ recurrent distrust in the FDA due to 4 main contributing factors: (1) that the FDA is influenced by the tobacco, agricultural, and pharmaceutical industries; (2) that the FDA is influenced by money and politics; (3) that the FDA is a bureaucracy exercising monopoly and power; and (4) that the FDA lacks technical capacity and competence to regulate tobacco products^16^.

Fourth, there are considerable disparities in e-cigarette use of lesbian, gay, bisexual, or transgender (LGBT) people, particularly those who are at the same time racial and ethnic minority^37^. In a study, e-cigarette use prevalence was higher for most non-heterosexual youths from racial and ethnic minority backgrounds than their heterosexual counterparts. However, multivariable logistic analysis showed varied results by racial and ethnic groups, with higher e-cigarette use odds for sexual minority youths, although not statistically significant for some racial and ethnic groups. African American gay or lesbian (OR: 3.86) and bisexual (OR: 3.31) high school students had significantly higher e-cigarette use odds than African American heterosexuals. Non-Hispanic African American females e-cigarettes use odds are 0.45 times that of non-Hispanic White males, and non-Hispanic other gay or lesbian had 3.15 times higher e-cigarette use odds than non-Hispanic white heterosexuals^37^.

Fifth, while White and higher-income people who smoke are likely to begin using e-cigarettes as a replacement for conventional cigarettes when they quit cigarettes, African American and low income individuals are likely to use e-cigarette without quitting cigarettes, with differences in e-cigarette uptake possibly partly explained by perceived harm or social norms of e-cigarettes^38^. If e-cigarettes in the US are seen as a replacement and harm reduction that is replacing cigarette, then the population that is not showing such a transition is still showing disparities. That means even lower use of e-cigarette becomes a sign of disparities in racial and ethnic minorities such as African Americans, even if there are some considerable risks in e-cigarette use^39^. Still, some level of privilege is needed for Americans to use e-cigarettes instead of conventional cigarettes, which is a type of injustice and a potential source of disparities. Perhaps the most notable finding in the Spears et al. study was an association that is generally consistent in the ENDS literature: people who smoke cigarettes and have greater resources and more social “privilege” (e.g., higher income and educational attainment or White race) adopt ENDS at higher rates. Emerging longitudinal evidence suggests that these groups are also more likely to switch to exclusive ENDS use, thereby reducing harm. Several factors may explain this pattern, including ENDS accessibility, cost, social norms, and risk perceptions. If this divergence persists, differential rates of quitting cigarettes by using ENDS may exacerbate smoking-related disparities. This would not be the first example of a disruptive technology or medical innovation that shifts the socioeconomic health gradient in favor of higher-resourced individuals^38,40^.

A number of policy changes^41^ are required to reverse the e-cigarette trends that have become a significant public health concern over the past decade^42^. Current regulations around e-cigarette marketing and sales are inadequate and do not sufficiently protect youths from exposure, access, and use of e-cigarettes^43^. Currently, many youths are exposed through social media. There are also policy debates around the effectiveness of various tax strategies for curbing youths’ e-cigarette use. Policies should discourage e-cigarette use while not encouraging replacement with combustible tobacco products as well as imposing limits on nicotine content in e-cigarettes and other tobacco products to minimize the risk of addiction and continued use. Currently, e-cigarette companies are in what has been called a “nicotine arms race [defined as rush to sell more and compete with each other]”^44-46^ with some products being sold providing nicotine doses far higher than others^47^. However, overall, nicotine pharmacokinetic curves for plasma nicotine levels from disposable, rechargeable, and closed tank e-cigarettes (these are the types used by the vast majority of people who use e-cigarettes) are MUCH lower compared to cigarettes^48^. In addition, the disease burden is shown to be lower for e-cigarettes than conventional cigarettes^49^. The high dose of nicotine in most vaping products, coupled with their unique formulation (nicotine salts) and the frequency in which it is inhaled relative to cigarette smoke, make these products highly addictive and very difficult for people to quit using once use has begun. Cessation via FDA-approved mechanisms such as nicotine replacement therapies may be challenging, because nicotine replacement therapies may have comparatively lower doses of nicotine, that are aimed at controlling cravings^44,50^.

Finally, policies that regulate point-of-sale marketing may restrict access of youths to e-cigarettes^51^. Restrictive regulations should ensure that these products do not target marginalized and disadvantaged communities in ways that the tobacco, alcohol, and cannabis industries have done for decades, to the detriment of minoritized and economically disadvantaged populations^52^. Rather than being sold in convenience stores, gas stations, or other venues that make them easily accessible to youths and ubiquitous in lower SES communities, or online where minors can easily bypass age restriction requirements, e-cigarettes intended as an alternative for adults to combustible tobacco products for cessation purposes should be available only behind the counter in pharmacies, using a model similar to that required for the sale of pseudoephedrine products^53,54^. Furthermore, companies interested in marketing e-cigarettes as smoking cessation tools should be required to use the established FDA process for bringing a new drug to market while assuring their safety and efficacy^55,56^.

Inadequate regulations and oversight of e-cigarettes since their emergence on the market over a decade ago have caused an astonishingly high number of middle and high school students and young adults, many of whom otherwise would not have been susceptible to nicotine use, to use a highly addictive product. Specific restrictive and regulatory policies related to e-cigarette sales^51,57,58^ and advertisement^59,60^ can help to better address this public health problem and assure that the potential benefits of e-cigarettes are delivered in a manner that does not harm a significant portion of the population (Figure 2).

In addition to policy measures, community-based participatory initiatives should prioritize educating young individuals about the risks associated with e-cigarettes and offering assistance to conventional tobacco users in their efforts to quit, including the use of e-cigarettes if necessary. Given the limited reach of healthcare systems and policies to marginalized populations, coupled with the challenges these communities face in terms of trust and accessibility, it is crucial to actively involve and engage with these communities. As an example, the Communities Engaged and Advocating for a Smoke-Free Environment (CEASE) program^61-65^, initially centered on addressing issues related to conventional cigarettes, has proven to be a successful initiative based on Community-Based Participatory Research (CBPR) principles and can be adapted effectively for ENDS as well^61-65^. Collaborating with community organizations with historic trust in the community, such as Amplify and the African American Tobacco Control Leadership Council (AATCLC), can indeed be a valuable asset for various initiatives and goals. Such partnerships can be beneficial in numerous ways, including: (a) Building Credibility and Trust: These organizations often have established relationships and trust within the community. Their involvement can lend credibility to your initiative, as community members are more likely to trust and engage with organizations they already know and respect. (b) Local Knowledge: Community organizations have an in-depth understanding of the community’s needs, culture, and challenges. They can provide valuable insights that help tailor your initiative to better address these specific issues. (c) Reaching Target Audiences: These organizations can help you reach the right audiences effectively. They often have well-defined networks and communication channels within the community, making it easier to disseminate information and mobilize support. (d) Advocacy and Awareness: Community organizations can advocate for your cause and raise awareness. They can use their platforms and resources to promote the initiative, helping it gain visibility and support. (e) Cultural Sensitivity: These organizations can guide your initiative in being culturally sensitive. This is crucial when dealing with diverse communities, as a one-size-fits-all approach may not be effective. They can help you avoid cultural missteps and ensure that your messaging resonates with the community. (f) Resource Sharing: Collaborating with established organizations can also mean sharing resources, whether it’s physical resources, knowledge, or human capital. This can help in the efficient implementation of your initiative. (g) Bidirectional communication and feedback Community organizations can provide feedback and help with the evaluation of your initiative’s impact. They can offer valuable insights into what is working and what needs improvement, allowing for continuous refinement. (h) Mobilizing Community Support: These organizations can assist in mobilizing community members to actively participate in the initiative, whether through volunteering, advocacy, or other means. It is very important to approach these partnerships with respect, humility, and a willingness to listen to the insights and recommendations of the community organizations. By working in collaboration you can create a more effective and community-centered initiative (Table 1).

Despite the inherent logic of many policy recommendations to curb exposure, access, and use of e-cigarettes among young people and those disproportionately targeted by their marketing and sale, the effectiveness of specific policy interventions must be tested prior to implementation. We need to conduct research to evaluate the effectiveness of various policy and regulatory strategies and promote and advocate for those most likely to protect public health.

National efforts can be made to increase population knowledge regarding e-cigarette risk. There are successful models with promising results. Education of the public should start with youths so initiation is prevented^66^. Perception and knowledge can be improved so the uptake of e-cigarettes in youths are lower^67^.

Before concluding this piece, several crucial points need additional clarification. It is essential to recognize that this is not an exhaustive or a systemic review but rather an expression of the author’s opinions regarding intersections of e-cigarette, marginalization, and tobacco control policies. It is important to use sensitive language when we write or advocate for vulnerable populations or any policies^68^. Caution is also warranted because there remain several unanswered questions that have not been addressed within this piece. It is of utmost importance to maintain an impartial perspective and carefully evaluate the evidence from both sides of the argument – those against e-cigarettes and those in favor of them. As electronic cigarettes deliver nicotine in an aerosol form that is likely to be substantially less harmful than cigarette smoke^69^ it might be more effective at helping smokers quit smoking cigarettes than nicotine replacement therapy^70,71^.

The evidence is still inconclusive on whether e-cigarettes are a safe replacement for conventional cigarettes. Although it may be safer^72^, many adults have the perception that e-cigarettes are less safe than conventional cigarettes^73^. This is still a field with large controversies and different beliefs. For example, one may argue that tobacco use was coming down and this trend was disrupted by the increasing trend of electronic cigarette usage. Other researchers have suggested that one of the reasons tobacco and conventional cigarette usage has declined is that many youths who would have used conventional cigarettes use electronic cigarettes instead^74^. Many adults already believe that e-cigarettes are as or more harmful compared to cigarettes, as shown by HINTS survey data^73^: Researchers should address the unintended consequence of adult misperceptions about e-cigarette harms. In addition, cigarette use among youth has decreased from 2019-2022, e.g. compare the NYTS 2019 results^74^ to NYTS 2022 results^75^: Current (past 30-day) e-cigarette use decreased by more than half among middle and high school students from 20.0% in 2019^74^ to 9.4% in 2022^75^. Thus, while inadequate regulations and oversight of e-cigarettes since their emergence on the market over a decade ago has seen considerable use of e-cigarettes by young people, the prevalence of e-cigarette use among middle and high school students has decreased by more than 50% in recent years^74,75^.

In a recent analysis of the Population Assessment of Tobacco and Health (PATH) Study data, a multistate transition model of 28,061 adults in Waves 4–5 (2017–19) and 24,751 adults in Waves 5–6 (2019–21) investigated transition rates for initiation, cessation, and switching products for each period overall and by age group. Authors found that although the persistence of ENDS use among adults remained high in 2019–21, a larger fraction of dual users transitioned to ENDS-only use. The study also showed only a small fraction of cigarette-only users switch to dual use and the risk of dual use of ENDS and conventional cigarettes are not on rise^76^. This added^77,78^ to the existing information regarding transition of epidemiology of ENDS use.

Another issue is the unintended consequences of our policies. Here is a hypothetical scenario: pharmacies are less accessible to those in lower SES communities, which means that removing e-cigarettes from convenience stores/gas stations will prevent lower SES people who smoke from accessing these potentially life-saving products with which they can switch away from the much more harmful behavior of smoking. That would increase disparities in tobacco burden among lower SES people. An example is the experience of Australia, where a prescription-only model was implemented and had disastrous results. Australia has some of the highest rates of youth e-cigarette use and an enormous illicit market of e-cigarettes sold illegally on streets with no regulatory oversight. As a majority of physicians incorrectly believe that nicotine is carcinogenic^79^ (although nicotine is not itself carcinogen), very few physicians would prescribe e-cigarette to patients (as is the case in Australia)^80^. In short, policymakers should consider the unintended consequences of any model such as the prescription-only model. Such policies may lead to increased disparities and more tobacco-related death and disease.

This short piece cannot address all the complex issues around ENDS. We cited and referred to evidence from both sides of the argument. For instance, when considering the existing evidence, such as the superior efficacy of e-cigarettes in aiding tobacco cessation compared to nicotine replacement therapies, as reported in the Cochrane review, it becomes evident that e-cigarettes may still serve as a valuable harm reduction tool for adults. A balanced examination of the arguments concerning the potential harm and benefits of e-cigarettes should continue to be a topic of debate. It is crucial not to overlook the primary role of e-cigarettes as a harm reduction tool for tobacco users. Nonetheless, it is important to acknowledge that most risks are associated with dual usage and initiation of e-cigarette use due to accessibility concerns.

In conclusion, although many scholars have avoided using the term disparities for e-cigarette, we need to rethink this issue. We believe there are considerable non-traditional disparities when it comes to e-cigarettes. Fundings and investments should be available to tackle e-cigarettes in vulnerable populations such as African American and Hispanic, dual users, non-quitters, those marginalized by policies, Native Americans, and LGBT people. More restrictive policies are needed to reduce the burden of e-cigarettes.

## Figures and Tables

**Figure 1:**
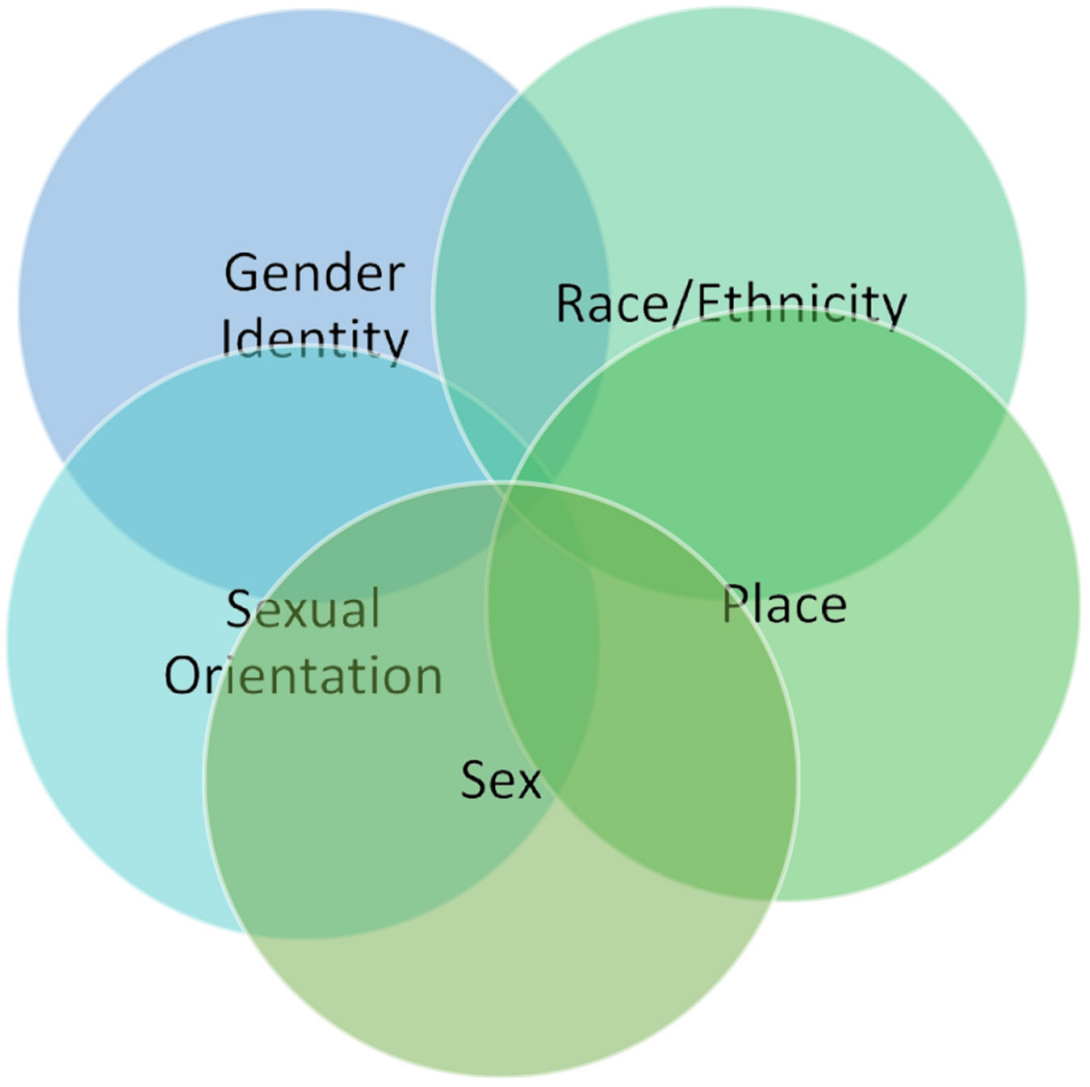
Intersections of marginalizing identities may be associated with prevalence of ENDS use.

**Figure 2:**
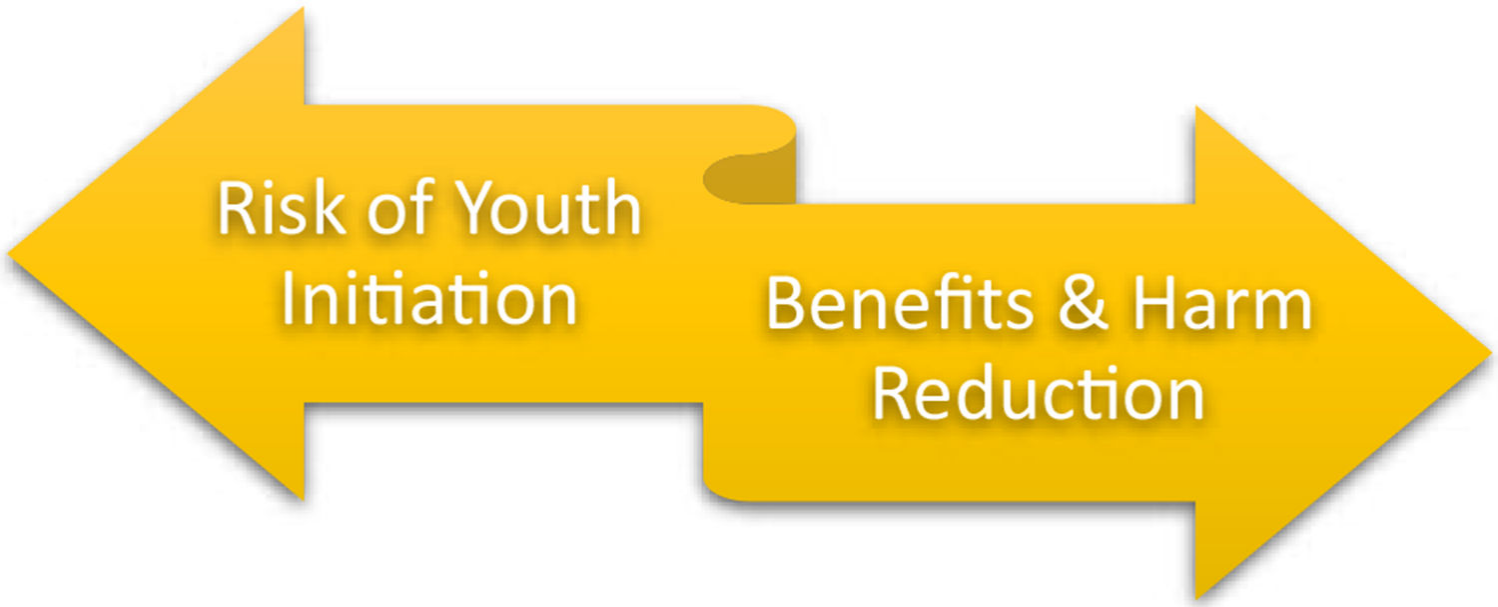
The major dilemma is to find policies that maximize ENDS utility as a harm reduction tool without risking youth and young adults.

**Table 1:** Benefits of using community-based participatory research (CBPR) approach

(a) Credibility and Trust
(b) Local Knowledge
(c) Reaching Target Audiences
(d) Advocacy and Awareness
(e) Cultural Sensitivity
(f) Resource Sharing
(g) Bidirectional communication and feedback
(h) Mobilizing Community Support

## References

[1] Jankowski M, Krzystanek M, Zejda JE, E-cigarettes are more addictive than traditional cigarettes—a study in highly educated young people. International journal of environmental research and public health. 2019; 16(13): 2279 31252671 10.3390/ijerph16132279 PMC6651627

[2] Azagba S, Baskerville NB, Minaker L. A comparison of adolescent smoking initiation measures on predicting future smoking behavior. Preventive medicine reports. 2015; 2:174–177. 26844068 10.1016/j.pmedr.2015.02.015 PMC4721447

[3] Legleye S, Janssen E, Beck F, Social gradient in initiation and transition to daily use of tobacco and cannabis during adolescence: a retrospective cohort study. Addiction. 2011; 106(8): 1520–1531. 21631617 10.1111/j.1360-0443.2011.03447.x

[4] Marques P, Piqueras L, Sanz M-J. An updated overview of e-cigarette impact on human health. Respiratory research. 2021; 22(1): 1–14. 34006276 10.1186/s12931-021-01737-5 PMC8129966

[5] Livingston JA, Chen C-H, Kwon M, Physical and mental health outcomes associated with adolescent E-cigarette use. Journal of pediatric nursing. 2022; 64:1–17. 35121206 10.1016/j.pedn.2022.01.006

[6] Huang J, Duan Z, Kwok J, Vaping versus JUULing: how the extraordinary growth and marketing of JUUL transformed the US retail e-cigarette market. Tobacco control. 2019; 28(2): 146–151. 29853561 10.1136/tobaccocontrol-2018-054382 PMC6274629

[7] Leventhal AM, Madden DR, Peraza N, Effect of exposure to e-cigarettes with salt vs free-base nicotine on the appeal and sensory experience of vaping: a randomized clinical trial. JAMA network open. 2021; 4(1): e2032757–e2032757. 33433597 10.1001/jamanetworkopen.2020.32757 PMC7804919

[8] Hammond D, Reid JL, Burkhalter R, Trends in e-cigarette brands, devices and the nicotine profile of products used by youth in England, Canada and the USA: 2017–2019. Tobacco control. 2023; 32(1): 19–29. 34099572 10.1136/tobaccocontrol-2020-056371 PMC9359003

[9] Dai H, Ramos AK, Faseru B, Racial disparities of e-cigarette use among US youths: 2014–2019. American journal of public health. 2021; 111(11): 2050–2058. 34554815 10.2105/AJPH.2021.306448 PMC8630507

[10] Dai H, Leventhal AM. Prevalence of e-cigarette use among adults in the United States, 2014-2018. Jama. 2019; 322(18): 1824–1827. 31524940 10.1001/jama.2019.15331 PMC6749536

[11] Moss SL, Keyes KM. Commentary on Foxon & Selya (2020): Social gradients in long-term health consequences of cigarette use—will adolescent e-cigarette use follow the same trajectory? Addiction (Abingdon, England). 2020; 115(12): 2379. 33047826 10.1111/add.15268 PMC9125695

[12] Hartwell G, Thomas S, Egan M, E-cigarettes and equity: a systematic review of differences in awareness and use between sociodemographic groups. Tobacco control. 2017; 26(e2): e85–e91. 28003324 10.1136/tobaccocontrol-2016-053222 PMC5739861

[13] Benny C, Steele BJ, Patte KA, Income inequality and daily use of cannabis, cigarettes, and e-cigarettes among Canadian secondary school students: Results from COMPASS 2018–19. International Journal of Drug Policy. 2023; 115: 104014. 37003193 10.1016/j.drugpo.2023.104014

[14] Kirchner TR, Villanti AC, Cantrell J, Tobacco retail outlet advertising practices and proximity to schools, parks and public housing affect Synar underage sales violations in Washington, DC. Tobacco Control. 2015; 24(el): e52–e58. doi:10.1136/tobaccocontrol-2013-051239 24570101

[15] Mead EL, Cruz-Cano R, Bernat D, Association between Florida’s smoke-free policy and acute myocardial infarction by race: A time series analysis, 2000–2013. Preventive Medicine. 2016; 92: 169–175. doi:10.1016/j.ypmed.2016.05.032 27261406 PMC6071670

[16] Smiley SL, Blackman KCA, Bluthenthal RN, “Who’s Really Regulating? Who’s Benefiting?” Exploring Black Stakeholders’ Awareness and Trust in the Food and Drug Administration’s Role as a Tobacco Regulator. Tob Regul Sci. 2018; 4(4): 41–49. doi:10.18001/trs.4.4.5 PMC6705425 31440525

[17] Alcalá HE, Sharif MZ, Morey BN. Misplaced Trust: Racial Differences in Use of Tobacco Products and Trust in Sources of Tobacco Health Information. Nicotine Tob Res. 2017; 19(10): 1199–1208. doi:10.1093/ntr/ntx080 28387825 PMC6580933

[18] Health UDo, Services H. Smoking cessation: a report of the Surgeon General. Atlanta: US Department of Health and Human Services. 2020. 32255575

[19] Webb Hooper M, Kolar SK. Racial/ethnic differences in electronic cigarette knowledge, social norms, and risk perceptions among current and former smokers. Addict Behav. 2017; 67: 86–91. doi:10.1016/j.addbeh.2016.12.013 28063324

[20] Seo YS, Chang YP. Racial and Ethnic Differences in E-Cigarette and Cigarette Use Among Adolescents. J Immigr Minor Health. 2022; 24(3): 713–720. doi:10.1007/s10903-021-01229-0 34106360

[21] Hu M-C, Davies M, Kandel DB. Epidemiology and Correlates of Daily Smoking and Nicotine Dependence Among Young Adults in the United States. American Journal of Public Health. 2006; 96(2): 299–308. doi:10.2105/ajph.2004.057232 16380569 PMC1470478

[22] Control CfD, Prevention. Health disparities related to commercial tobacco and advancing health equity.

[23] Luo Z, Alvarado GF, Hatsukami DK, Race Differences in Nicotine Dependence in the Collaborative Genetic Study of Nicotine Dependence (COGEND). Nicotine & Tobacco Research. 2008; 10(7): 1223–1230. doi:10.1080/14622200802163266 18629733

[24] Kathryn CE, Karin AK, Zhiqun T, Correlates of tobacco product reuptake and relapse among youth and adults in the USA: findings from the PATH Study Waves 1–3 (2013–2016). Tobacco Control. 2020; 29(Suppl 3): s216. doi:10.1136/tobaccocontrol-2020-055660 32321855 PMC7517708

[25] Hillier A, Chilton M, Zhao Q-W, Peer reviewed: Concentration of tobacco advertisements at SNAP and WIC stores, Philadelphia, Pennsylvania, 2012. Preventing Chronic Disease. 2015. 10.5888/pcd12.140133 PMC4318686 25654220

[26] Primack BA, Bost JE, Land SR, Volume of tobacco advertising in African American markets: systematic review and meta-analysis. Public Health Reports. 2007; 122(5): 607–615. 17877308 10.1177/003335490712200508 PMC1936959

[27] Widome R, Brock B, Noble R The relationship of neighborhood demographic characteristics to point-of-sale tobacco advertising and marketing. Ethnicity & health. 2013; 18(2): 136–151. 22789035 10.1080/13557858.2012.701273

[28] Clark PA. A legacy of mistrust: African-Americans, the medical profession, and AIDS. The Linacre Quarterly. 1998; 65(1): 66–88. 11660554 10.1080/00243639.1998.11878407

[29] White RM. Misinformation and misbeliefs in the Tuskegee Study of Untreated Syphilis fuel mistrust in the healthcare system. Journal of the National Medical Association. 2005; 97(11): 1566. 16334509 PMC2594917

[30] Lee MJ, Reddy K, Chowdhury J, Overcoming the legacy of mistrust: African Americans’ mistrust of medical profession. 2018.

[31] Mayer M, Reyes-Guzman C, Grana R, Demographic Characteristics, Cigarette Smoking, and e-Cigarette Use Among US Adults. JAMA Netw Open. 2020; 3(10): e2020694. doi:10.1001/jamanetworkopen.2020.20694 33048127 PMC8094416

[32] Assari S, Mistry R, Bazargan M. Race, Educational Attainment, and E-Cigarette Use. J Med Res Innov. 2020. doi:10.32892/jmri.185 PMC7034862 32090188

[33] Rolle IV, Beasley DD, Kennedy SM, National Surveys and Tobacco Use Among African Americans: A Review of Critical Factors. Nicotine Tob Res. 2016; 18 Suppl l(Suppl 1): S30–40. doi:10.1093/ntr/ntv195 26980862 PMC6200134

[34] Garrett BE, Dube SR, Trosclair A, Cigarette smoking—united states, 1965–2008. MMWR Surveill Summ. 2011; 60(1): 109–113. 21430635

[35] Pronk NP, Kleinman DV, Richmond TS. Healthy People 2030:Moving toward equitable health and well-being in the United States. EClinicalMedicine. 2021; 33: 100777. doi:10.1016/j.eclinm.2021.100777 33733077 PMC7941044

[36] Holford TR, Levy DT, Meza R. Comparison of smoking history patterns among African American and white cohorts in the United States born 1890 to 1990. Nicotine & Tobacco Research. 2016; 18(suppl_1): S16–S29. 26980861 10.1093/ntr/ntv274 PMC5009449

[37] Azagba S, Ebling T, Shan L. Sexual Minority Youth E-Cigarette Use. Pediatrics. 2023; 151(3): e2022058414. doi:10.1542/peds.2022-058414 36808534

[38] Harlow AF, Stokes A, Brooks DR. Socioeconomic and Racial/Ethnic Differences in E-Cigarette Uptake Among Cigarette Smokers: Longitudinal Analysis of the Population Assessment of Tobacco and Health (PATH) Study. Nicotine Tob Res. 2019; 21(10): 1385–1393. doi:10.1093/ntr/nty141 29986109 PMC6751515

[39] Giovenco DP. Different Smokes for Different Folks? E-Cigarettes and Tobacco Disparities. Am J Public Health. 2019; 109(9): 1162–1163. doi:10.2105/ajph.2019.305250 31390250 PMC6687245

[40] Spears CA, Jones DM, Weaver SR, Sociodemographic Correlates of Electronic Nicotine Delivery Systems (ENDS) Use in the United States, 2016-2017. Am J Public Health. 2019; 109(9): 1224–1232. doi:10.2105/ajph.2019.305158 31318599 PMC6687271

[41] Harris JK, Moreland-Russell S, Choucair B, Tweeting for and against public health policy: response to the Chicago Department of Public Health’s electronic cigarette Twitter campaign. Journal of medical Internet research. 2014; 16(10): e238. 25320863 10.2196/jmir.3622 PMC4210950

[42] Cullen KA, Gentzke AS, Sawdey MD, E-cigarette use among youth in the United States, 2019. Jama. 2019; 322(21): 2095–2103. 31688912 10.1001/jama.2019.18387 PMC6865299

[43] Diaz MC, Donovan EM, Schillo BA, Menthol e-cigarette sales rise following 2020 FDA guidance. Tobacco control. 2021; 30(6): 700–703. 32967985 10.1136/tobaccocontrol-2020-056053

[44] Leelavathi L. Nicotine Replacement Therapy for Smoking Cessation-An Overview. Indian Journal of Public Health Research & Development. 2019.

[45] Richtel M. E-cigarette makers are in an arms race for exotic vapor flavors. New York Times. 2014.

[46] Jones AC, Felton GW, Tumlinson JH. The dual function of elicitors and effectors from insects: reviewing the ‘arms race’against plant defenses. Plant molecular biology. 2022:1–19. 10.1007/s11103-021-01203-2 34618284

[47] Goniewicz ML, Gupta R, Lee YH, Nicotine levels in electronic cigarette refill solutions: A comparative analysis of products from the US, Korea, and Poland. International Journal of Drug Policy. 2015; 26(6): 583–588. 25724267 10.1016/j.drugpo.2015.01.020 PMC4457636

[48] Fearon IM, Eldridge AC, Gale N, Nicotine pharmacokinetics of electronic cigarettes: A review of the literature. Regulatory Toxicology and Pharmacology. 2018; 100: 25–34. doi:10.1016/j.yrtph.2018.09.004 30201538

[49] Oh AY, Kacker A. Do electronic cigarettes impart a lower potential disease burden than conventional tobacco cigarettes?: Review on e-cigarette vapor versus tobacco smoke. The Laryngoscope. 2014; 124(12): 2702–2706. doi:10.1002/lary.24750 25302452

[50] Lindson N, Chepkin SC, Ye W, Different doses, durations and modes of delivery of nicotine replacement therapy for smoking cessation. Cochrane Database of Systematic Reviews. 2019; 4(4): CD013308. 30997928 10.1002/14651858.CD013308 PMC6470854

[51] Klein DE, Chaiton M, Kundu A, A literature review on international e-cigarette regulatory policies. Current Addiction Reports. 2020; 7: 509–519.

[52] Brown-Johnson CG, England LJ, Glantz SA, Tobacco industry marketing to low socioeconomic status women in the USA. Tobacco control. 2014; 23(e2): e139–e146. 24449249 10.1136/tobaccocontrol-2013-051224 PMC4105326

[53] Gaiha SM, Lempert LK, Halpern-Felsher B. Underage youth and young adult e-cigarette use and access before and during the coronavirus disease 2019 pandemic. JAMA network open. 2020; 3(12): e2027572–e2027572. 33270127 10.1001/jamanetworkopen.2020.27572 PMC7716191

[54] Do EK, Aarvig K, Donovan EM, Underage youth continue to obtain E-cigarettes from retail sources in 2022: evidence from the truth continuous tracking survey. International Journal of Environmental Research and Public Health. 2023; 20(2): 1399. 36674152 10.3390/ijerph20021399 PMC9859475

[55] Lipsky MS, Sharp LK. From idea to market: the drug approval process. The Journal of the American Board of Family Practice. 2001; 14(5): 362–367. 11572541

[56] Dabrowska A, Thaul S. How FDA approves drugs and regulates their safety and effectiveness. Washington: Congressional Research Service. 2018:1–25.

[57] Chen-Sankey J, Cruz-Cano R, Pakdaman S, Associations between living in localities with e-cigarette sales restrictions and e-cigarette use change among young adults in Los Angeles County. Tobacco control. 2022; 31(Suppl 3): sl87–sl96. 10.1136/tc-2022-057478 PMC9639823 36328463

[58] Schiff S, Liu F, Cruz TB, E-cigarette and cigarette purchasing among young adults before and after implementation of California’s tobacco 21 policy. Tobacco control. 2021; 30(2): 206–211. 32108084 10.1136/tobaccocontrol-2019-055417 PMC8260105

[59] Owotomo O, Walley S. The youth e-cigarette epidemic: updates and review of devices, epidemiology and regulation. Current problems in pediatric and adolescent health care. 2022; 52(6): 101200. 35577717 10.1016/j.cppeds.2022.101200

[60] D’Angelo H, Patel M, Rose SW. Convenience store access and e-cigarette advertising exposure is associated with future e-cigarette initiation among tobacco-naive youth in the PATH study (2013–2016). Journal of Adolescent Health. 2021; 68(4): 794–800. 10.1016/j.jadohealth.2020.08.030 PMC8317228 33039271

[61] Apata J, Oladele A, Fahimi S, Monday-Enhanced CEASE Program for Underserved Ethnic Minorities: a Mixed-Methods Study. J Racial Ethn Health Disparities. 2023: 1–15. doi:10.1007/s40615-023-01570-0 PMC10062259 36995578

[62] Sheikhattari P, Apata J, Bleich L, Efficacy of a Smoking Cessation Program for Underserved Ethnic Minority Communities: Results of a Smoking Cessation Trial. Int J Public Health. 2023; 68: 1605739. doi:10.3389/ijph.2023.1605739 37408795 PMC10318133

[63] Sheikhattari P, Apata J, Kamangar F, Examining Smoking Cessation in a Community-Based Versus Clinic-Based Intervention Using Community-Based Participatory Research. J Community Health. 2016; 41(6): 1146–1152. doi:10.1007/s10900-016-0264-9 27688221 PMC5083217

[64] Apata J, Sheikhattari P, Bleich L, Addressing Tobacco Use in Underserved Communities Through a Peer-Facilitated Smoking Cessation Program. J Community Health. 2019; 44(5): 921–931. doi:10.1007/s10900-019-00635-8 30843139 PMC6708456

[65] Apata J, Sheikhattari P, Bleich L, Addressing Tobacco Use in Underserved Communities Through a Peer-Facilitated Smoking Cessation Program. Journal of Community Health. 2019; 44(5): 921–931. doi:10.1007/s10900-019-00635-8 30843139 PMC6708456

[66] Gaiha SM, Duemler A, Silverwood L, School-based e-cigarette education in Alabama: Impact on knowledge of e-cigarettes, perceptions and intent to try. Addictive Behaviors. 2021; 112:106519. 32890911 10.1016/j.addbeh.2020.106519

[67] Gibson LA, Creamer MR, Breland AB, Measuring perceptions related to e-cigarettes: important principles and next steps to enhance study validity. Addictive Behaviors. 2018; 79: 219–225. 29175027 10.1016/j.addbeh.2017.11.017 PMC5807230

[68] Hefler M, Durkin SJ, Cohen JE, New policy of people-first language to replace ‘smoker’,’vaper’’tobacco user’and other behaviour-based labels. BMJ Publishing Group Ltd. 2023:133–134. 10.1136/tc-2023-057950 PMC9985717 36806099

[69] Hartmann-Boyce J, Butler AR, Theodoulou A, Biomarkers of potential harm in people switching from smoking tobacco to exclusive e-cigarette use, dual use or abstinence: secondary analysis of Cochrane systematic review of trials of e-cigarettes for smoking cessation. Addiction. 2023; 118(3): 539–545. 36208090 10.1111/add.16063 PMC10092879

[70] Hartmann-Boyce J, Begh R, Aveyard P. Electronic cigarettes for smoking cessation. Bmj. 2018. 10.1136/bmj.j5543 29343486

[71] Hartmann-Boyce J, Lindson N, Butler AR, Electronic cigarettes for smoking cessation. Cochrane database of systematic reviews. 2022. 10.1002/14651858.CD010216.pub4 PMC8094228 33052602

[72] Rom O, Pecorelli A, Valacchi G, Are E-cigarettes a safe and good alternative to cigarette smoking? Annals of the New York Academy of Sciences. 2015; 1340(1): 65–74. 25557889 10.1111/nyas.12609

[73] Bjurlin MA, Basak R, Zambrano I, Perceptions of e-cigarette harm among cancer survivors: Findings from a nationally representative survey. Cancer Epidemiology. 2022; 78: 102037. doi:10.1016/j.canep.2021.102037 34561186

[74] Wang TW, Gentzke AS, Creamer MR, Tobacco product use and associated factors among middle and high school students—United States, 2019. MMWR Surveillance Summaries. 2019; 68(12): 1. 10.15585/mmwr.ss6812a1 PMC6903396 31805035

[75] Park-Lee E, Ren C, Cooper M, Tobacco product use among middle and high school students—United States, 2022. Morbidity and Mortality Weekly Report. 2022; 71(45): 1429–1435. 36355596 10.15585/mmwr.mm7145a1 PMC9707354

[76] Brouwer AF, Jeon J, Jimenez-Mendoza E, Changing patterns of cigarette and ENDS transitions in the USA: a multistate transition analysis of adults in the PATH Study in 2017–2019 vs 2019–2021. medRxiv. 2023. doi:10.1101/2023.10.20.23297320

[77] Brouwer AF, Jeon J, Hirschtick JL, Transitions between cigarette, ENDS and dual use in adults in the PATH study (waves 1–4): multistate transition modelling accounting for complex survey design. Tobacco control. 2022; 31(3): 424–431. 10.1136/tobaccocontrol-2020-055967 PMC8124082 33199541

[78] Brouwer AF, Jeon J, Jimenez-Mendoza E, Changing patterns of cigarette and ENDS transitions in the USA: a multistate transition analysis of youth and adults in the PATH Study in 2015–2017 vs 2017–2019. Tobacco Control. 2023. 10.1136/tc-2022-057905 PMC10533746 36977570

[79] Steinberg MB, Bover Manderski MT, Wackowski OA, Nicotine Risk Misperception Among US Physicians. Journal of General Internal Medicine. 2021; 36(12): 3888–3890. doi:10.1007/s11606-020-06172-8 32875504 PMC8642586

[80] Cohen JE, Gartner C, Edwards R, Australia tightens its prescription-only regulation of e-cigarettes. British Medical Journal Publishing Group. 2023. 10.1136/bmj.p1216 37280000

